# Calls to Action (Mobilizing Information) on Cancer in Online News: Content Analysis

**DOI:** 10.2196/26019

**Published:** 2021-06-21

**Authors:** Thomas Hongjie Zhang, Jen Sern Tham

**Affiliations:** 1 Department of Communication Faculty of Modern Languages and Communication Universiti Putra Malaysia Serdang Malaysia

**Keywords:** mobilizing information, online cancer news, quantitative content analysis, Malaysia, online news, cancer, infodemiology, media, digital media, digital health, health information, cancer health information

## Abstract

**Background:**

The health belief model explains that individual intentions and motivation of health behaviors are mostly subject to external cues to action, such as from interpersonal communications and media consumptions. The concept of mobilizing information (MI) refers to a type of mediated information that could call individuals to carry out particular health actions. Different media channels, especially digital media outlets, play an essential role as a health educator to disseminate cancer health information and persuade and mobilize cancer prevention in the community. However, little is known about calls to action (or MI) in online cancer news, especially from Asian media outlets.

**Objective:**

This study aimed at analyzing cancer news articles that contain MI and their news components on the selected Malaysian English and Chinese newspapers with online versions.

**Methods:**

The Star Online and Sin Chew Online were selected for analysis because the two newspaper websites enjoy the highest circulation and readership in the English language and the Chinese language streams, respectively. Two bilingual coders searched the cancer news articles based on sampling keywords and then read and coded each news article accordingly. Five coding variables were conceptualized from previous studies (ie, cancer type, news source, news focus, cancer risk factors, and MI), and a good consistency using Cohen kappa was built between coders. Descriptive analysis was used to examine the frequency and percentage of each coding item; chi-square test (confidence level at 95%) was applied to analyze the differences between two newspaper websites, and the associations between variables and the presence of MI were examined through binary logistic regression.

**Results:**

Among 841 analyzed news articles, 69.6% (585/841) presented MI. News distributions were unbalanced throughout the year in both English and Chinese newspaper websites; some months occupied peaks (ie, February and October), but cancer issues and MI for cancer prevention received minimal attention in other months. The news articles from The Star Online and Sin Chew Online were significantly different in several news components, such as the MI present rates (χ^2^=9.25, *P*=.003), providing different types of MI (interactive MI: χ^2^=12.08, *P*=.001), interviewing different news sources (government agency: χ^2^=12.05, *P*=.001), concerning different news focus (primary cancer prevention: χ^2^=10.98, *P*=.001), and mentioning different cancer risks (lifestyle risks: χ^2^=7.43, *P*=.007). Binary logistic regression results reported that online cancer news articles were more likely to provide MI when interviewing nongovernmental organizations, focusing on topics related to primary cancer prevention, and highlighting lifestyle risks (odds ratio [OR] 2.77, 95% CI 1.89-4.05; OR 97.70, 95% CI 46.97-203.24; OR 186.28; 95% CI 44.83-773.96; *P*=.001, respectively).

**Conclusions:**

This study provided new understandings regarding MI in cancer news coverage. This could wake and trigger individuals’ preexisting attitudes and intentions on cancer prevention. Thus, health professionals, health journalists, and health campaign designers should concentrate on MI when distributing health information to the community.

## Introduction

Cancer is a pressing public health issue and does not discriminate whether in a developed or developing country [1,2]. Cancer, in fact, remains the second leading cause of death throughout the world. The World Health Organization [1] reported that in 2018, 9.6 million people were estimated to have died from cancer. In other words, 1 in 6 deaths is from cancer of some type. Lung, prostate, colorectal, stomach, and liver cancer are the most common types of cancers in men, while breast, colorectal, lung, cervical, and thyroid cancer are the most common among women [1]. According to a recent report from the International Agency for Research on Cancer [3], breast cancer occupied the first leading incidence rate in Malaysia (17.3%), followed by colorectal cancer (14.0%) and lung cancer (10.7%). Local cancer incidence was not equal across ethnic groups. In recent years, Malaysian Chinese have had the highest cancer incidence rate for the majority of leading cancers. It is more elevated than Malays and Indians [4,5].

Scientific findings showed that at least half of all cancers occurring today could be prevented if individuals adopted healthy lifestyles and behaviors, and many other cancers can be diagnosed through medical interventions in the early stages [6]. Hence, it is crucial to promote cancer prevention and early detection to the individuals. Essentially, cancer prevention is no longer treated as a health issue alone but a developmental issue for a nation. Health promotion regarding cancer issues involves collaborations from different stakeholders, which include conventional media and digital media.

The media provides various health resources [7]. In reality, media can be a vital health educator, bridging the information gap between health care practitioners and the public in cancer communication [8,9]. The media publicize activities or campaigns from medical or governmental sectors and translate medical language and other jargon into public language [10,11]. Nowadays, besides health professionals, the internet has become a popular source of health information [12]. It changes the way an individual makes health decision, perceives health issues, and interacts with health professionals [13]. Cancer-related topics are the most commonly searched health topics online [14], and cancer news coverage is one of the most consumed health news categories by online newsreaders [15]. The majority of online news sites contain specific sections for conveying health news coverage: it allows information-oriented news consumers and active health information seekers to obtain timely health information about issues that concern them [16,17].

The health belief model explains that individual intentions and motivation of health behaviors are mostly subject to external cues, such as interpersonal communications and media consumptions [18]. It was defined as a variable called cue to action [19]. Based on this viewpoint, Tanner and Friedman [20] linked the concept of mobilizing information (MI) onto the theoretical ground of the health belief model. Originally, MI was a type of information widely examined by scholars from political science and journalism studies [21,22]. By definition, in the realm of public health, MI refers to a type of mediated information that could call individuals to carry out particular health actions (eg, quit smoking) or trigger them to act on preexisting attitudes effectively. Preexisting attitudes involve one’s overall evaluation on a subject matter which later forms preconceived notions even if they did not experience it personally [23]. Since health information from media channels is usually persuasive, it acts as an actuator for health behavior change [7]: when individuals encounter persuasive health information, especially MI, their cognition and memories related to the particular health issue would be evocated and, in turn, brought to further actions. Specifically, MI includes the venue, time, and contact information of a health campaign, detailed educational information of how to a prevent certain disease or reduce risks, and additional links to provide further readings or interactions [20,24]. The individuals could actively or passively engage with different types of health news content that contain a certain type of MI. Thus, MI can reach newsreaders easily during their routine news consumption.

Several studies have examined MI in different health news coverage, but previous researchers found that there was very limited MI presented in the news content. For example, one study found that there was only approximately 30% of chronic disease coverage presented MI in Canadian aboriginal newspapers [25]. Another study reported that only 3 in 10 online health news articles from American local television websites presented MI [20]. Moreover, previous studies treated MI as an affiliated coding variable, scrutinizing on the present rate or types of MI instead of investigating the in-depth relationships between MI and other news components, such as news source and news focus. Also, most of the past studies examining MI are from the Western and English media channels [26,27]; little is known about how the Eastern and non-English media provide MI in health news coverage.

In Malaysia, media channels practice multilingual media policy to serve different communities [28]; the characteristics of the local media industry differ from other countries. Even in the local Chinese society, the media preference varies based on the socioeconomic background or other factors [28]. Thus, it is research-worthy for analyzing and comparing the local media contents published in different languages. Scholars reported that the way the media cover cancer news depends highly on who the target audience is [29]. Therefore, the focal points of the approach of presenting MI on cancer issues might differ from the local English media and the Chinese media. Since Malaysian Chinese populations had the highest cancer incidence rate among 3 main ethnicities [5], it is vital to know how Malaysian English and Chinese online news sites cover cancer issues and how these news sites mobilize individuals to prevent cancer. To fill the abovementioned research gaps, this study aims to answer the following research questions:

- RQ1a: What are the present rates, patterns, and types of MI in cancer news coverage on an English news site and a Chinese news site?
- RQ1b: Are there differences in the present rates, patterns, and types of MI on cancer coverage between an English news site and a Chinese news site?
- RQ2a: What are the characteristics of news components (ie, cancer type, news sources, news focus, and cancer risk factors) in cancer news coverage that provided MI on an English news site and a Chinese news site?
- RQ2b: Are there differences in the characteristics of news components (ie, cancer type, news sources, news focus, and cancer risk factors) in cancer news coverage that provided MI on an English news site and a Chinese news site?
- RQ3: Are there associations in the cancer coverage presenting MI between news sources, news focus, and cancer risk factors on an English news site and a Chinese news site?

## Methods

In this study, we applied quantitative content analysis to examine cancer news articles that contain MI on selected Malaysian English and Chinese newspapers with online versions and compare the differences between the newspaper websites.

### Selection of Content Units

According to the Reuters Institute Digital News Report 2019 [30], The Star Online occupied 29% of the local weekly online news media use, which ranked the highest among all English newspaper websites. Sin Chew Online occupied 12% of it, which ranked the highest score among all local Chinese newspaper websites.

**Figure 1:**
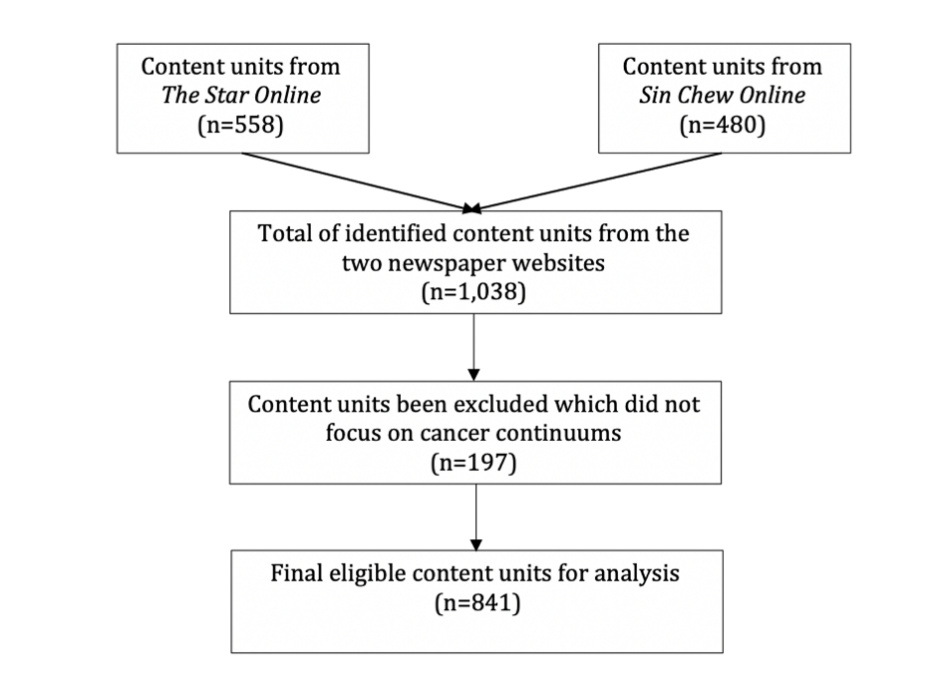
Process of selecting and excluding content units.

The unit of analysis of this study was the news article. News articles were retrieved using a keyword search of the homepages of individual newspaper websites. The keywords such as “cancer” OR “cancer prevention” OR “cancer treatment” OR “breast cancer” OR “lung cancer” OR “prostate cancer” OR “colon cancer,” etc, appearing in the headlines, subheadlines, first 3 paragraphs and lead paragraphs were used to find articles about MI on cancer prevention. We excluded news articles that covered obituaries of public figures who died from cancer and did not focus on cancer treatment or suggestions for cancer prevention. A total of 841 news articles were identified from 2017 to 2019. The process of selecting and excluding news articles for analysis is shown in Figure 1.

### Research Instrument and Descriptions

Interrelated coding book and coding sheet were predesigned for the data collection purpose. There are 5 variables in the coding instruments that were conceptualized based on previous studies. There are several options under each variable; the number of options that should be chosen depends on the measurement and operationalization of each variable. All the variables were identified as nominal measurements. The operational definitions of each coding variable are shown in Multimedia Appendix 1.

#### Description of Cancer Type

Two categories of cancers (Multimedia Appendix 2) were examined in this study (ie, highly preventable cancers and highly detectable cancers). This description was adopted from Moriarty and Stryker’s categorization of cancers [31], which was approved by the American Cancer Society. If an article covered any cancer-related topic without focusing on a specific type of cancer or covered more than one particular type of cancer, such as coverage on cancer care facilities’ improvement, cancer-related health policy change, or statistic report on incidence rate/mortality rate, the article was then coded as cancer in general. Coders were requested to identify one type of cancer for a news article only.

#### Description of News Source

News source has a vital impact on health news coverage, and it is also a crucial variable for content analysis [32]. The description of news source in this study was adopted from past studies [33,34]. Six types of news sources were examined: medical journal, research institution, pharmaceutical company, government agency, nongovernmental organization (NGO), and other. Coders coded more than one news source for an article if applicable.

#### Description of News Focus

Different news articles cover an issue through different foci. The description of news focus was based on Tanner and Friedman’s study [20]. There were 6 types of news focus under this study (ie, primary cancer prevention, secondary cancer prevention, medical treatment, social support/survivorship, medical research, and statistical report). Coders coded more than one type of news focus for an article if applicable.

#### Description of Cancer Risk Factors

This description aimed to identify the types of cancer risks mentioned in cancer news coverage. There were four categories of cancer risk factors that could be identified from cancer news articles, which are lifestyle risks, environmental/occupational risks, demographical risks, and medical risks. It was conceptualized and used in previous studies [35,36]. Coders coded more than one cancer risk factor for an article if applicable.

#### Description of Mobilizing Information

This description is the primary research focus of this study and includes 2 coding items (presence of MI and types of MI). First, the presence of MI in the news article was identified. Coders identified whether each article presented MI by coding present or absent in the coding sheet. If MI is absent from the article, the coders needed not to code the types of it.

If MI is presented in the article, then coders identified the types of MI in the article. There are 4 types of MI defined by previous research [20,21]: locational MI (ie, location of a hospital or event), identificational MI (ie, contact details of a health care provider), tactical MI (ie, suggestion or educational information regarding how to prevent cancer), and interactive MI (ie, hyperlinks for further readings). Coders coded more than one type for an article if applicable.

### Intercoder Reliability

Two coders (the first author and a graduate student) randomly coded 10% of the identified news articles for intercoder reliability check. The graduate student received extensive training before coding the articles. We employed a Cohen kappa reliability test [37]. Based on the Altman strength of agreement, Cohen kappa value ranging from .61 to .80 indicates good agreement while values falling from .81 to 1.0 show very good agreement [38]. Our results demonstrated that some coding items obtained good agreement, such as tactical MI (k=.73, 95% CI .59-.87) and medical cancer risks (k=.74, 95% CI .59-.88), and the rest of items gained very good agreement between 2 coders, like cancer type (k=.89, 95% CI .78-.98) and presence of MI (k=.94, 95% CI .85-1.0).

### Data Analysis

The obtained data were analyzed via SPSS (version 26.0, IBM Corp). First, we applied descriptive analysis and chi-square test to answer RQ1 and RQ2, including the frequencies, percentages, and differences of each news component. We then employed binary logistic regression (forward likelihood ratio method) to determine the associations between different news components and the presence of MI in online cancer news (RQ3). The level of significance in this study was set as *P*<.05.

## Results

There were 585 out of 841 cancer news articles on The Star Online and Sin Chew Online provided at least one type of MI in a span of 36 months from January 2017 to December 2019 (Table 1). The overall present rate of MI was 69.6% (585/841), which means nearly 7 in 10 of cancer news articles provided MI. Specifically, 64.4% (283/436) of articles on The Star Online, and 74.6% (302/405) of news articles on Sin Chew Online provided MI. The present rate of MI on Sin Chew Online was higher than The Star Online (χ^2^=9.25, df=1; *P*=.003).

**Table 1:** The presence of mobilizing information in cancer news coverage on The Star Online and Sin Chew Online (2017-2019; n=841).

MI^a^ presence	The Star Online, n (%)	Sin Chew Online, n (%)	Overall, n (%)
Present	283 (64.4)	302 (74.6)	585 (69.6)
Absent	152 (35.6)	103 (25.4)	258 (30.4)
Total	436 (100)	405 (100)	841 (100)

^a^MI: mobilizing information.

**Figure 2:**
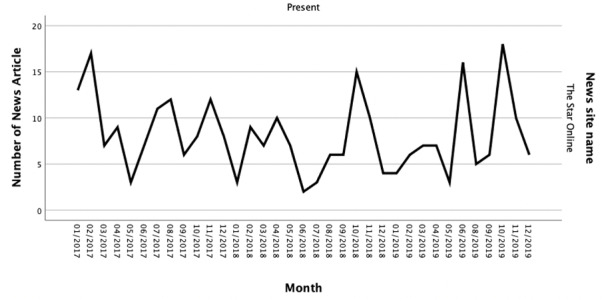
Numbers of cancer news articles provided mobilizing information on The Star Online from Jan 2017 to Dec 2019.

**Figure 3:**
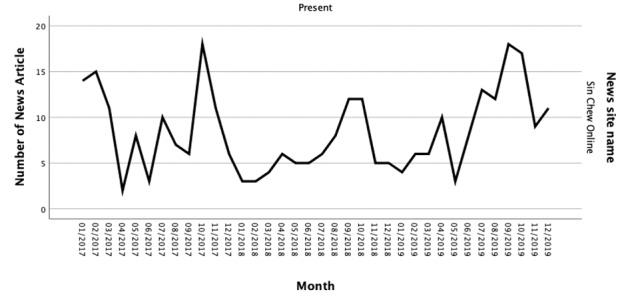
Numbers of cancer news articles provided mobilizing information on Sin Chew Online from Jan 2017 to Dec 2019.

The overall patterns of monthly distribution showed a fluctuation in both newspaper websites (Figures 2 and 3). The patterns of cancer news articles providing MI between the 2 news sites were slightly different. For The Star Online, the peak was in October 2019, and there were 18 cancer news articles contained MI. However, no cancer news article provided MI in July 2019. Sin Chew Online had 2 peak months, October 2017 and September 2019. There were 18 cancer news articles delivered MI in both months. The lowest month for Sin Chew Online was April 2017, which only had 2 cancer news articles that provided MI.

For the types of MI (Table 2), the majority of news articles (419/585, 71.6%) from both news sites provided tactical MI. Locational MI was the second most frequently presented in the news articles, which occupied 25.1% (109/585). Besides, there were similar patterns on providing identificational MI (96/585, 16.4%) and interactive MI (107/585, 18.3%), both provided by less than 20% of cancer news articles. The chi-square results indicated that news articles in The Star Online provided more interactive MI (χ^2^_1_=12.08, *P*=.001), while news articles in Sin Chew Online provided more identificational MI (χ^2^_1_=5.44, *P*=.03).

**Table 2:** Distributions of types of mobilizing information (n=585).

Type of MI^a^	The Star Online, n (%)	Sin Chew Online, n (%)	Overall, n (%)	χ^2^	*P* value
Locational MI	91 (15.6)	109 (18.6)	200 (34.2)	1.0	.34
Identificational MI	36 (6.1)	60 (10.3)	96 (16.4)	5.4	.03
Tactical MI	192 (32.8)	227 (38.8)	419 (71.6)	3.9	.05
Interactive MI	68 (11.6)	39 (6.7)	107 (18.3)	12.1	.001

^a^MI: mobilizing information.

Descriptive analysis and chi-square test for each news component were conducted to answer RQ2a and RQ2b (Table 3). More than half of the cancer news articles that provided MI did not focus on a specific cancer (313/585, 53.5%), and there were overall 36.1% (211/585) of news articles focused on highly detectable cancers such as breast cancer, prostate cancer, liver cancer, and stomach cancer. Only 10.4% (61/585) of articles gave attention to highly preventable cancers such as lung cancer and cervical cancer. There was a significant difference between the websites in covering different cancer types (χ^2^_1_=8.05, *P*=.02).

Pertaining to news sources, more than half (333/585, 57%) of the cancer news articles that provided MI from both newspaper websites interviewed staff from medical institutions, followed by NGOs (208/585, 35.5%). Less than 20% of the news articles interviewed cancer patients, cancer survivors, or other individuals who did not belong to any institution (102/585, 17.5%) or government agency (97/585, 16.6%). Very few news articles cited medical journals (37/585, 4.7%) or interviewed pharmaceutical companies (8/585, 1%). The chi-square results indicated that there was a significant difference between newspaper websites and news sources used while reporting cancer news. (11.3% [66/585] vs 5.3% [31/585]; χ^2^_1_=12.05, *P*=.001). Specifically, Sin Chew Online skewed to refer to government agencies as the source in reporting cancer news.

As for news focus, the majority (410/585, 70%) of cancer news articles that provided MI focused on primary cancer prevention and almost half (285/585, 48.7%) focused on secondary cancer prevention. In addition, 28.0% news articles that focused on social support contained MI (164/585), followed by medical treatment (127/585, 21.7%), medical research (80/585, 13.7%), and statistical reports on cancer issues (69/585, 11.8%). The chi-square results showed that news articles in The Star Online were more likely to focus on medical treatment (13% [76/585] vs 8.7% [51/585]; χ^2^_1_=8.54, *P*=.004); news articles in Sin Chew Online were more likely to look at primary cancer prevention (39.3% [230/585] vs 30.8% [180/585]; χ^2^_1_=10.98, *P*=.001).

In terms of cancer risk factors, 63.0% (368/585) of cancer news articles that provided MI mentioned demographical risks, followed by medical risks (281/585, 48.1%) and lifestyle risks (236/585, 40.4%), respectively. Only 3.3% (19/585) of news articles gave attention to environmental/occupational risks that may cause cancer. The chi-square result showed that news articles in Sin Chew Online focused more on lifestyle risks than articles in The Star Online (23.6% [138/585] vs 16.8% [98/585]; χ^2^_1_=7.43, *P*=.007).

**Table 3:** Descriptive analysis of news components in cancer news coverage that provided mobilizing information (n=585).

Variable and category		The Star Online, n (%)		Sin Chew Online, n (%)		Overall, n (%)		χ^2^		*P* value
**Cancer type**		—^a^		—		—		8.1		.02
	General cancer topic	139 (23.8)	174 (29.7)		313 (53.5)		—		—	
	Highly preventable cancer	39 (6.7)	22 (3.8)		61 (10.5)		—		—	
	Highly detectable cancer	105 (17.9)	106 (18.1)		211 (36.0)		—		—	
**News source**		—		—		—		—		—
	Medical journal	21 (3.6)	16 (2.7)		37 (6.3)		1.1		.31	
	Medical institution	173 (29.6)	160 (27.4)		333 (57.0)		4.0		.06	
	Pharmaceutical company	5 (0.9)	3 (0.5)		8 (1.4)		0.7		.50	
	Government agency	31 (5.3)	66 (11.3)		97 (16.6)		12.1		.001	
	Nongovernmental organization	105 (17.9)	103 (17.6)		208 (35.5)		0.6		.49	
	Other individuals	53 (9.1)	49 (8.4)		102 (17.5)		0.4		.45	
**News focus**		—		—		—		—		—
	Primary cancer prevention	180 (30.8)	230 (39.3)		410 (70.0)		11.0		.001	
	Secondary cancer prevention	149 (25.5)	136 (23.2)		285 (48.7)		3.4		.07	
	Medical treatment	76 (13.03)	51(8.7)		127 (21.7)		8.5		.004	
	Social support	77 (13.2)	87 (14.8)		164 (28.0)		0.2		.71	
	Medical research	47 (8.0)	33 (5.6)		80 (13.6)		4.0		.05	
	Statistical report	34 (5.8)	35 (6.0)		69 (11.8)		0.03		.09	
**Cancer risk factors**		—		—		—		—		—
	Lifestyle risks	98 (16.8)	138 (23.6)		236 (40.4)		7.4		.007	
	Environmental/occupational risks	8 (1.4)	11 (1.9)		19 (3.3)		0.3		.65	
	Demographic risks	184 (31.5)	184 (31.5)		368 (63.0)		1.1		.35	
	Medical risks	142 (24.3)	139 (23.8)		281 (48.1)		1.0		.32	

^a^Not applicable.

A binary logistic regression analysis (forward likelihood ratio method) was conducted to answer RQ3. Table 4 presents these results accordingly. First, the results indicated that news articles that interviewed NGOs and medical institutions were more likely to present MI (OR 2.77, 95% CI 1.89-4.05; OR 1.85, 95% CI 1.33-2.58, respectively). News articles that cited medical journals as the source were also more likely to present MI (OR 2.28, 95% CI 1.03-5.01), but the significant level was lower than NGOs and medical institutions (95% [*P*=.04] vs 99% [*P*=.001]). Therefore, NGOs, being the news source, obtained the strongest association with the presence of MI. There was no significant association found for other news sources, such as medical journals, pharmaceutical companies, or governmental agencies in the examined news articles.

Second, for news focus, the binary logistic regression results reported that news articles were more likely to present MI when they focused on primary cancer prevention (OR 97.70, 95% CI 46.97-203.24) and secondary cancer prevention (OR 22.12, 95% CI 12.16-40.22). However, news articles that focused on medical research were less likely to present MI (OR .33, 95% CI .12-.37). The equation model shows that articles that focus on primary cancer prevention obtained the strongest association with the presence of MI. Other news focus such as medical treatment, social support/survivorship, or statistical report were not significantly associated with the presence of MI.

In terms of cancer risk factors, the binary logistic regression results showed that all 4 types of cancer risk factors (ie, lifestyle, environmental/occupational, demographic, and medical) were positively associated with the presence of MI in the online cancer news. However, the significant levels varied. The news articles that reported lifestyle risks obtained the strongest association with the presence of MI (OR 186.28; 95% CI 44.83-773.96), followed by demographic risks (OR 8.97; 95% CI 6.08-12.25), and medical risks (OR 3.07; 95% CI 2.08-4.53). The news articles that reported environmental/occupational risks were also associated with the presence of MI (OR 3.83; 95% CI 1.15-12.76). However, when comparing to the other 3 risk categories, the significance level of the association between environmental/occupational risks and the presence of MI was relatively weak (only at 95%).

**Table 4:** Associations between news sources, news focus, cancer risk factors, and the presence of mobilizing information (n=841).

Variable and step		Category	OR^a^ (95% CI)	*P* value
**News source^b^**				
	1	Nongovernmental organization	1.97 (1.40-2.77)	.001
	2	Nongovernmental organization	2.69 (1.84-3.93)	.001
		Medical institution	1.91 (1.37-2.67)	.001
	3	Nongovernmental organization	2.77 (1.89-4.05)	.001
		Medical institution	1.85 (1.33-2.58)	.001
		Medical journal	2.28 (1.03-5.01)	.04
**News focus^c^**				
	1	Primary cancer prevention	64.30 (312.31-129.97)	.001
	2	Primary cancer prevention	91.05 (44.55-186.09)	.001
		Secondary cancer prevention	23.32 (12.96-41.95)	.001
	3	Primary cancer prevention	97.70 (46.97-203.24)	.001
		Secondary cancer prevention	22.12 (12.16-40.22)	.001
		Medical research	.33 (0.12-0.37)	.001
**Cancer risk factors^d^**				
	1	Lifestyle risks	85.88 (21.15-384.65)	.001
	2	Lifestyle risks	138.12 (33.63-567.37)	.001
		Demographic risks	8.52 (5.88-12.35)	.001
	3	Lifestyle risks	180.50 (43.54-748.36)	.001
		Demographic risks	8.50 (5.80-12.49)	.001
		Medical risks	2.94 (2.00-4.32)	.001
	4	Lifestyle risks	186.28 (44.83-773.96)	.001
		Demographic risks	8.97 (6.08-12.25)	.001
		Medical risks	3.07 (2.08-4.53)	.001
		Environmental/occupational risks	3.83 (1.15-12.76)	.03

^a^OR: odds ratio.

^b^The −2 log likelihood = 997.94, Cox & Snell *R^2^*=.04, Nagelkerke *R^2^*=.06.

^c^The −2 log likelihood = 476.57, Cox & Snell *R^2^*=.48, Nagelkerke *R^2^*=.69.

^d^The −2 log likelihood = 659.42, Cox & Snell *R^2^*=.36, Nagelkerke *R^2^*=.51.

## Discussion

### Principal Findings

As the media has been instrumental in creating public awareness about cancer and chronic disease prevention, understanding the media’s role in mobilizing the public regarding cancer prevention is of utmost importance. This study provides the first comprehensive and comparative assessment of MI in cancer news on the selected Malaysian English and Chinese newspapers with online versions. Of importance, our results show that there were nearly 70% of the online cancer news articles in The Star Online and Sin Chew Online presented at least one type of MI. It indicates that the selected Malaysian newspaper websites have an awareness of mobilizing and calling health behavior change for its readers. However, our findings are contrary to previous studies that examined MI in cancer coverage in Western countries [22,24-26]. Several possible explanations could support our findings. First, since the past studies that examined MI in cancer news coverage were conducted at least 10 years ago, the patterns of cancer news coverage have already changed, especially for online news media [22,24-26]. Second, Hoffman [20] introduced another type of MI called interactive MI, which is only applicable for online news content. This could be one of the reasons why online cancer news contained more MI as compared to print media. Third, the increasing rates of cancer incident and mortality around the world [1], the seriousness of the disease, health information needs among the people, and the media attention of cancer issues that differ from the past have become another dependable rationale to support our results. Fourth, culture and social background would give impacts to health coverage [39]. Thus, our results report that the characteristics of cancer news coverage in the selected Malaysian newspaper websites were quite different from the Western countries. Of note, we also found that cancer news articles in Sin Chew Online were more likely to present MI than those in The Star Online. This may due to the increased cancer incident rate reported among the Chinese population [4,5], and Sin Chew Online realized that it is crucial to promote and mobilize cancer prevention to its targeted readers.

Our results illustrated that there was an unbalanced monthly distribution in terms of the number of cancer news articles that provided MI. In certain months, there were more cancer news articles found providing MI, such as in February, October, and November. This may due to the fact that World Cancer Day falls in February, Children’s Cancer Awareness Month falls in September, and Breast Cancer Awareness Month falls in October. These occasions gained more media attention because of shared journalistic values; journalists usually cover health topics according to the allocation of relevant campaigns or events [40]. In the same vein, Varga and colleagues [40] reported that the media coverage on certain cancers mushroomed during their awareness months, such as breast cancer and prostate cancer, and especially on social media platforms. However, in certain months, there were fewer cancer news articles reporting MI, such as in June and July. These months are the busier months throughout the year as there are many local festivals and school holidays happen in these months. The Hari Raya Aidilfitri, Gawai Dayak, King’s birthday, and Dragon Boat Festival happen in these 2 months. Negative news such as reporting cancer issues and related death tolls would likely give way to positive news and announcements such as big sales and discounts, tourism, and Open House. Hence, the news distribution of MI on cancer prevention was found less in June and July. Speaking of the types of MI, we found that the majority of cancer news provided tactical MI, which contained how-to health information and detailed suggestions about cancer prevention. Such news provides a clear picture to the readers of how to prevent cancers in their daily life.

We found several differences between The Star Online and Sin Chew Online regarding news components providing MI in online cancer news. First, the results indicated that there was a significant difference in terms of cancer types portrayed in the 2 studied online news websites. More than half of cancer news that provided MI focused more than one specific type of cancer. It mainly covered health campaigns or events related to cancer prevention, followed by highly detectable cancer and highly preventable cancer. This phenomenon is consistent with the previous study, which applied the same categorization of cancer types [31]. By comparison, cancer news from Sin Chew Online focused more on general cancer topics while The Star Online is more likely to cover highly preventable cancer issues.

For news source, the results showed that the majority of cancer coverage that provided MI interviewed professionals from medical institutions, such as public and private hospitals, universities or laboratories, followed by NGOs. This corroborates with previous studies that examined sourcing practices in various health coverage [41,42]. Interestingly, we found that cancer news articles that provided MI in The Star Online were more likely to cite medical journals from overseas countries, while those in Sin Chew Online were more likely to interview government agencies and NGOs from the local Chinese society, such as ancestral connective organizations and religious organizations serve for Chinese communities (ie, Chinese temples and Chinese-dominant churches). This shows that cancer coverage from different languages has varying source preferences subject to their journalistic values and social/cultural norms [39].

Pertaining to news focus, most cancer news providing MI focused on primary cancer prevention, such as local educational events or social marketing campaigns related to cancer issues, followed by secondary cancer prevention, like mobilizations on cancer screening for early detection and other medical interventions. Since the characteristics of MI include triggers and call the readiness among individuals, it is reasonable that the majority of cancer coverage that provided MI focused on prevention, rather than other topics like cancer treatment or cancer research [20]. We found that cancer news providing MI in The Star Online focused slightly more on topics related to secondary cancer prevention and medical treatment as compared to the coverage reported in Sin Chew Online, which was more likely to concentrate on primary cancer prevention and social support topics. This phenomenon reflects Malaysian media ecology when it comes to health news coverage.

As for cancer risk factors, our results showed different findings compared to the previous study where this description was adopted [35]. More than half of the examined news coverage mentioned demographic risks, like cancer-related with a specific gender or age. Most of these demographic risks pointed to female cancers, such as cervical cancer prevention, HPV vaccination, and breast cancer self-checking. Almost half of cancer news articles mentioned medical risks and lifestyle risks when providing MI. It corroborates the dominant characteristics of MI that promotes behavioral change on health issues [20], which advocates changes and adoptions of health behavior, as well as an understanding of medical knowledge and preventive strategies among the readers. However, it is worth noting that there was rarely cancer coverage that provided MI on environmental risks, such as natural resources pollutions, radiations, and occupational risks. The reason could be due to lack of knowledge on environmental issues and their association with cancer morbidity and mortality [43]. By comparing the two newspaper websites, we found that cancer news from Sin Chew Online was more likely to mention lifestyle risks when providing MI. It may subject to the cancer incidence in local Chinese communities; reducing risk lifestyle is one of the dominant approaches to avoid cancer occurrence [5,6].

The logistic regression analysis unveiled the in-depth findings of this study. This method is inspired by previous studies that used the same approach to analyze other media messages [34,44]. We examined the associations between 3 critical news components (ie, news sources, news focus, and cancer risk factors) and the presence of MI in online cancer news separately. For the news sources, our results illustrated that the cancer news articles were most likely to present MI when NGOs were interviewed as the source, followed by medical institutions and medical journals. This result agrees with a previous study finding that elite sources are the dominant news sources in health news coverage [32]. This study suggests that health journalists should be more active in engaging with NGOs and medical institutions. On this count, NGOs could bring up-to-date resources for events and campaigns regarding cancer prevention, and medical institutions can provide credible and applicable health information for cancer prevention.

A finding of interest was that MI was more likely to be presented in online cancer news when articles focused on primary cancer prevention, such as health education, consultation, health events or campaigns, followed by secondary cancer prevention, like promotions of early detection, screening, or diagnosis. On the other hand, MI was less likely to be presented when articles covered topics related to medical research. Thus, we recommend that health journalists should devote more attention on how to cover topics related to cancer education, local events, and campaigns, which would mobilize and persuade the newsreaders to take proactive actions to prevent cancer instead of merely providing medical research-related news retrieved from collaborative news agencies.

Regarding cancer risk factors, we found that even though every type of cancer risk was positively associated with the presence of MI, the probability level was different. The articles that reported lifestyle risks such as obesity, alcohol consumption, or smoking causing cancer were positively associated with the presence of MI in online cancer news. This is consistent with previous findings which showed that since various cancers occurring today are related to unhealthy lifestyles, cancer news coverage switched their major attention from highlighting environmental causes to discussing lifestyle causes [35]. Our results also showed that the mentions of demographic risks, like risks related to gender and age, were positively associated with the presence of MI. However, we found out that the majority of news articles that mentioned demographic risks pointed to females, which suggests that future events or campaigns could focus on mobilizing females to prevent breast and cervical cancer, as well as educating this target group to raise their awareness on cancers that most prevalent among females. There were limited news articles on demographic risks provided MI that targeted males even though prostate cancer and testicular cancer are two serious health issues for males [45]. We suggest that health journalists need to notice that cancer coverage should not skew to cancers related to females only. It is also essential to raise awareness and mobilize males to prevent cancers that are associated with them. The lack of coverage on cancers related to males calls for the need for research on effective strategies to reduce health information disparities among the male population.

### Limitations

This study contains some limitations. First, we only applied a keyword searching strategy for searching online news articles, and we might have missed some relevant news articles due to human errors. Future researchers should improve news sample selecting strategy by applying technology-based approaches to minimize human errors. Second, we only analyzed MI in online cancer news from the selected Malaysian newspaper websites. We did not consider the cancer news published in Malay and Tamil newspaper websites, which are two other major languages spoken in Malaysia. The representativeness of this study, hence, is limited. More studies should give attention to cancer news published in Malay and Tamil; it would draw a more comprehensive picture in terms of covering cancer issues and providing MI in Malaysian media. Third, we determined the original definition of tactical MI is ambiguous, which points to “the explicit and implicit instructions for certain behavior” [19,20]. The understanding and operationalization of this concept highly depend on different researchers and newsreaders. Thus, communication scholars need to argue and re-theorize this definition in future studies. Fourth, it is worth noting that there is a lot of variation in the products and content of the agencies used as sources, such as their stature, professional staff, mission, and other characteristics that would explain or influence the variance in data collected. In this study, the researchers only categorized the sources in generic forms. Future studies could apply textual analysis, examining the latent content of news coverage or case study to explore these variations in detail. Fifth, this study did not compare the differences or correlations of representation of MI on the internet, print, and broadcast media. Hence, future research could examine whether if different types of MI are presented differently on different mediums. Last, our results only could represent the way of presenting MI by the online news media, but the effects of MI toward individuals are unknown. Future studies should examine the effects of MI and behavioral intention or outcomes among newsreaders.

### Conclusion

This study is one of the first scholarly attempts to throw light on analyzing and comparing MI of cancer news on selected English and Chinese newspaper websites. The findings and discussions corroborate new practical, theoretical, and methodological knowledge in health communication and journalism that would help public health researchers, medical professionals, health journalists, health policymakers, and NGOs in their researches and decision makings. The comprehensive picture of this study shows the characteristics and in-depth relationships between MI and different news components. For future studies, researchers could focus on online cancer news in other languages and also compare the differences between those published in native/national language and English. Also, experimental designs should be developed to examine the effects of MI on cancer prevention among different individuals. Furthermore, our methodological use could be applied in future studies that examine other variables in health news coverage, such as frame building and supplemental information.

## Appendix

Multimedia Appendix 1 Definitions of coding items.

Multimedia Appendix 2 Classifications of cancer type.

## Abbreviations

MI: mobilizing information
NGO: nongovernmental organization
OR: odds ratio
