# Supplementary material for: Calls to Action (Mobilizing Information) on Cancer in Online News: Content Analysis
Source: J Med Internet Res. 2021 Jun 21;23(6):e26019. doi: 10.2196/26019 (PMC8277372; doi:10.2196/26019)
Supplement: Multimedia Appendix 1 [file jmir_v23i6e26019_app1.docx]

**MULTIMEDIA APPENDIX 1**

***Definitions of Coding Items***

| **Coding Item** | **Definition** |
| --- | --- |
| ***News Type*** |  |
| Medical Finding News | The news article focused on the recent research findings related to cancer prevention or cancer treatment. |
| Educational News | The news article provided educational or persuasive information on cancer issues, which could initially enhance health awareness and trigger behavioural change for the readers, it could be an interview with a cancer care professional who provides credible health information. |
| Health Policy News | The news article covered health policy making/changing related to cancer issues, such as medical insurance on cancer or public service related to cancer care; the potential keywords could be “the Ministry of Health” and “government”. |
| Health Event News | The news article covered cancer prevention event/campaign launched in the community, such as a campaign for a particular type of cancer, social marketing program, public health seminar, or cancer awareness activity. |
| Promotional/Commercial News | The news article covered information for profitable products or services, like the introduction of a nutrition supplement or a medical service that helpful for cancer prevention. |
| Personal story | The news article covered cancer treatment experience of a cancer survivor or a famous public figure who is suffering from cancer. |
| others | The news article covered other types of news, which is related to cancer continuums but could not be codified as these types above. |
| ***News Source*** |  |
| Medical Journal | The news article cited research findings from a medical journal. |
| Medical Institution | The news article interviewed or cited the researcher, doctor or health professional from a research institution, a hospital or a university. |
| Pharmaceutical Company | The news article interviewed a pharmacist, a nutritionist, or a profitable healthcare provider from a private company. |
| Government Agency | The news article interviewed a governmental official from The Ministry of Health, or other federal or state governmental sectors. |
| NGOs | The news article interviewed the staff or cited the report from an NGO, such as the World Health Organisation (WHO) and the National Cancer Society Malaysia (NCSM). |
| Other Individuals | The news article interviewed or cited other sources which do not belong to any of the categories above, such as cancer patient, family member of cancer patient, and other individuals. |
| ***News Focus*** |  |
| Primary Cancer Prevention | The news article focused on cancer health consultation, health education or environmental risk factors control. |
| Secondary Cancer Prevention | The unit of news article focused on medical interventions such as detections, screenings, and effective diagnosis approaches for the specific type of cancer |
| Medical Treatment | The news article focused on medical treatment, such as an introduction for cancer medicine, a vaccine or a therapy approach which are already utilised in clinical treatment |
| Social Support/Survivorship | The news article focused on social support/survivorship, either a campaign points to financial help, an event or an activity launched by the government, private sectors, or NGOs to attract the social attention on cancer prevention. |
| Medical Research | The news article focused on recent alternative cancer research, which may introduce a novel way to prevents cancer cause or a new medical technology relates to cancer detection, but the research findings have not been applied in the clinical treatment yet. |
| Statistical Report | The unit of coding focused on statistic report on cancer incidence rate or mortality rate. |
| ***Cancer Risk Factors*** |  |
| Lifestyle Risks | The news article mentioned the risks related to a daily lifestyle that may cause cancer, such as alcohol consumption, smoking behaviour, physical inactive, sexual behaviour, sun exposure, obesity and mobile phone usage. |
| Environmental/Occupational risks | The news article mentioned the risks related to the environment where the people working or living may cause cancer, such as natural resources pollutions, chemical dangerous and dangerous working environment. |
| Demographical Risks | The news article mentioned the risks related to gender, ethnicity, ageing, and socioeconomic status that may be associated with cancer occurrence |
| Medical Risks | The news article mentioned the risks related to genetic reasons, specific medicine intake could increase the risk of cancer occurrence, or particular surgery or treatment for another disease that initially causes cancer. |
| ***Mobilising Information*** | A type of public affair information, which could call the individuals to behave on a particular action or trigger the public to act on their pre-existing attitude regarding cancer issues. |
| ***Type of MI*** |  |
| Locational MI | The news article mentioned the location for a cancer care hospital or the venue and the time for a cancer prevention-related campaign/event. |
| Identificational MI | The unit of coding mentioned the contact details (name and telephone number) of a cancer care professional, a campaign manager, or other governmental/private staff who are taking part in cancer care and cancer prevention. |
| Tactical MI | The news article provided “how-to” information or appropriate strategies/tips regarding cancer prevention. |
| Interactive MI | The news article included hyperlinks linked to another relevant website, video or comment section, as well as emails address, which can provide additional reading for the readers. |
