# Supplementary material for: Calls to Action (Mobilizing Information) on Cancer in Online News: Content Analysis
Source: J Med Internet Res. 2021 Jun 21;23(6):e26019. doi: 10.2196/26019 (PMC8277372; doi:10.2196/26019)
Supplement: Multimedia Appendix 2 [file jmir_v23i6e26019_app2.docx]

**MULTIMEDIA APPENDIX 2**

***Clasifications of Cancer Type***

| Type | Examples |
| --- | --- |
| **Highly Preventable Cancer** | - lung cancer - oesophageal cancer - bladder cancer - skin cancer |
| **Highly Detectable Cancer** | - breast cancer - cervical cancer - prostate cancer - colon/rectum cancer - stomach cancer - oral cancer - bone cancer - liver cancer - leukaemia - lymphoma |
| **Cancer in General** | - Coverage on cancer care facilities’ improvement; - Coverage on cancer health policy change; - Coverage on relevant general education information; - Coverage on statistic report; or - The news article mentioned more than one type of cancer. |
